# Using Text Mining Techniques to Identify Health Care Providers With Patient Safety Problems: Exploratory Study

**DOI:** 10.2196/19064

**Published:** 2021-07-27

**Authors:** Iris Hendrickx, Tim Voets, Pieter van Dyk, Rudolf B Kool

**Affiliations:** 1 Centre for Language Studies, Centre for Language and Speech Technology Faculty of Arts Radboud University Nijmegen Netherlands; 2 Dutch Health and Youth Care Inspectorate Utrecht Netherlands; 3 IQ healthcare Radboud Institute for Health Sciences Radboud University Medical Center Nijmegen Netherlands

**Keywords:** text mining, risk management, health care quality improvement

## Abstract

**Background:**

Regulatory bodies such as health care inspectorates can identify potential patient safety problems in health care providers by analyzing patient complaints. However, it is challenging to analyze the large number of complaints. Text mining techniques may help identify signals of problems with patient safety at health care providers.

**Objective:**

The aim of this study was to explore whether employing text mining techniques on patient complaint databases can help identify potential problems with patient safety at health care providers and automatically predict the severity of patient complaints.

**Methods:**

We performed an exploratory study on the complaints database of the Dutch Health and Youth Care Inspectorate with more than 22,000 written complaints. Severe complaints are defined as those cases where the inspectorate contact point experts deemed it worthy of a triage by the inspectorate, or complaints that led to direct action by the inspectorate. We investigated a range of supervised machine learning techniques to assign a severity label to complaints that can be used to prioritize which incoming complaints need the most attention. We studied several features based on the complaints’ written content, including sentiment analysis, to decide which were helpful for severity prediction. Finally, we showcased how we could combine these severity predictions and automatic keyword analysis on the complaints database and listed health care providers and their organization-specific complaints to determine the average severity of complaints per organization.

**Results:**

A straightforward text classification approach using a bag-of-words feature representation worked best for the severity prediction of complaints. We obtained an accuracy of 87%-93% (2658-2990 of 3319 complaints) on the held-out test set and an F1 score of 45%-51% on the severe complaints. The skewed class distribution led to only reasonable recall (47%-54%) and precision (44%-49%) scores. The use of sentiment analysis for severity prediction was not helpful. By combining the predicted severity outcomes with an automatic keyword analysis, we identified several health care providers that could have patient safety problems.

**Conclusions:**

Text mining techniques for analyzing complaints by civilians can support inspectorates. They can automatically predict the severity of the complaints, or they can be used for keyword analysis. This can help the inspectorate detect potential patient safety problems, or support prioritizing follow-up supervision activities by sorting complaints based on the severity per organization or per sector.

## Introduction

We know since some time that information from patients can help improve the quality and safety of care [1]. Previous studies [2-4] using patient and client experiences with health care providers focused on reviews in which patients describe their positive and negative experiences. By analyzing patient complaints, health care providers can detect preventable patient safety issues with opportunities for improvement [5], enable organizational learning, and identify poor outcomes [6,7]. We also know that a small group of health care providers causes a major part of these complaints [8]. Identifying these providers with potential safety problems is important to significantly improve patient safety [9].

Regulatory bodies such as health care inspectorates already use negative patient experiences to identify health care providers with patient safety problems. In Australia, health complaints commissions predict the risks of individual doctors becoming the subject of repeated patient complaints [10]. In the Netherlands, the Health and Youth Care Inspectorate uses repeated negative ratings from patient rating websites as part of their supervision to predict risks [11,12]. Furthermore, the Dutch Health and Youth Care Inspectorate started a national contact point for health care complaints in 2014. All citizens can file complaints against a health care provider at this national contact point of the inspectorate over telephone, using an online web form, or via email. The complaints are categorized and manually judged based on severity by the inspectorate contact point. In the majority of the complaints, the contact point can offer straightforward advice to the patients by referring them a local complaints officer, patient counselor, or the relevant external dispute resolution body of the health care provider. Incoming complaints from the public are only passed on to the national health care inspectors when the complaints are deemed to be extremely serious such as major patient safety risks, fraud, misconduct, and sexual harassment. Approximately 13% of the complaints are triaged by inspectors [13]. All complaints are stored in a database, which is a relatively new source of information for the inspectorate. However, the database comprehends huge amounts of data and it is challenging to identify the potential safety problems in this heap of mostly unstructured information. Text mining can be defined as “analyzing patterns in text data to extract and discover actionable knowledge directly useful for task completion or decision-making, thus providing more direct task support for users” [14].

As incoming complaints are handled one by one by the contact point team members, certain providers may have received several complaints, which on their own are not sufficiently severe to warrant further inspection. However, a series of complaints may indicate safety problems. Given the successful use of machine learning techniques in extracting information from large quantities of data, the application of these techniques may be promising for identifying health care providers with patient safety problems in such complaint databases.

The aim of this study was to explore whether the application of text mining techniques on the complaints database of the Dutch Health and Youth Care Inspectorate could help identify health care providers with patient safety problems that may harm patients. These problems are especially within the scope of the Dutch inspectorate. We investigated whether we could support the inspectorate by using text mining techniques to automatically predict the severity of the complaints and conducting sentiment analysis to prioritize visiting health care providers with potential patient safety problems.

## Methods

### Data Collection and Preparation

Since 2014, the Dutch health care system has a national contact point for complaints about care designated by the Dutch Health and Youth Care Inspectorate. All the incoming complaints are registered in a specific database with structured information formats. Each entry contains several fields such as the personal details of the person filing the complaint and the targeted health care provider. In this study, we focused on the request fields, providing descriptions of the complaints filed by the citizens. Other fields are also manually added by the contact point members such as category labels, sector labels, priority labels, reporting dates, whether the complaints are sent to an inspector for triage, and action fields listing the actions taken by the inspectorate. In total, the database contains 22,509 complaints for the time period between 2014 and 2017. Complaints arrive at the inspectorate contact point via different media. Telephone calls are transcribed by a contact point team member as a written summary of the verbal complaint, whereas web forms are linked directly to the database fields. Email content is usually added to the request field, which may also include notes from possible follow-up contacts. Letters, constituting the minority media, are scanned to a digital format and a brief summary is added by the contact point team in the “short description” field. In 2017, two-thirds of the complaints were filed over telephone and a quarter using online web forms.

As the complaint data are highly sensitive, during our research, the data remained on a secure server of the inspectorate that was not connected to the Internet and could only be accessed via secure login. Therefore, we had to bring the text mining software to the data. We designed a ready-made research environment for the text mining experiments [15] in the form of a virtual machine that was then installed on the local secure server.

We extracted the complaints from the inspectorate’s database and attempted to remove all mentions of personal information such as telephone numbers, addresses, and names from the free-text fields containing the complaint descriptions using regular expressions. We used the fields with personal information to detect the names and numbers to remove from the free-text fields in a preprocessing step. Removing every name and address reference in a text automatically is practically infeasible [16], but most personal information was removed. The motivation for removing person names from the text was for privacy reasons and obtaining clean data for text mining. The removed unique names and numbers are uninformative attributes in the text mining process. As the data were always kept on the secure inspectorate server, privacy was already guaranteed. The study was assessed and approved by the Research Ethics Committee of the Radboud University Medical Center in the Netherlands. The ethics committee waived the request to approve the study, as it did not fall under the Medical Research Involving Human Subjects Act in the Netherlands (number 2020-7024).

### Severity Prediction

Some complaints are more severe than others and the concept of severity can be viewed as a sliding scale. Gillespie and Reader [17] have also been labeling severity in health care complaints. Their Healthcare Complaints Analysis Tool (HCAT) is a well-validated instrument for manual labeling of complaint severity. It is a taxonomy and coding scheme for manual annotation of health care complaints. Each complaint is analyzed based on seven problem categories (such as quality communication and safety), and the level of severity (low, medium, and high) is assessed. In this HCAT coding scheme, the problem severity is coded separately from the health care outcomes (harm). The HCAT tool is intended for complaint labeling by human experts. However, we practically conceptualized severity as a supervised machine learning task, namely learning predict the severity of unseen complaints from the manual judgments on the severity of complaints. As mentioned in the introduction, the majority of the complaints are handled by the contact point members by offering advice or by redirecting them to local complaint committees or counselors. Only potential major patient safety risks are redirected to the national health care inspectors. We investigated two variants of the manually assigned labels in the Dutch complaints database that can be considered indicators of severity. First, every complaint that was sent from the contact point to an inspector for triage (Triage) was considered severe. Second, using a more restrictive option, we only considered complaints to be severe when the inspectorate decided to take action (such as investigating the health care provider) based on the complaint (Decision). Approximately 13% percent of all the complaints were sent to the inspectorate for triage, and approximately 6% were further investigated after the triage. We experimented with these two indicators to determine whether we could automatically learn to predict severity based on the written content of the complaints with a supervised machine learning approach.

### Information Representation

An obvious use of automatic severity prediction is that such an automatic technique can be applied to the incoming digital complaints to rank them based on the predicted severity so that the contact point members can prioritize the most severe complaints. For incoming complaints, only the request field (description of the complaints filed by the citizens) is known, and we used that field as the main information source of the complaint description. We represented the information from the request field in five ways:

#### Bag-of-Words Representation

The simplest representation of the text in the request field is to create a bag-of-words representation that contains a list of n-grams (a sequence of n neighboring words). The value of n was varied from 1 (single word) to a maximum length of 3. We restricted the list of n-grams to only those n-grams that occur in at least 5 complaints. We experimented with two different weighting schemes (termed frequency–inverse document frequency weighting and log scaling) to determine the importance of the n-grams. We also varied the list size of the top selected most informative words.

#### Specified Keywords

Instead of a bag of words, one can also focus on a subset of keywords that are expected to be informative. We filtered words in the complaints by mapping them to the Linguistic Inquiry and Word Count (LIWC) dictionary designed for psycholinguistic research [18]. This is accomplished by extracting keywords from the text and grouping them into categories. Each of these categories corresponds to a particular concept. This can include grammatical concepts (whether a word is a pronoun, verb, noun, etc) as well as concepts such as emotional states, motivations, intentions, and thought processes [19]. The version used here was a Dutch version developed by Zijlstra et al [20] that has been independently verified. Mapping the keywords resulted in a total of 64 features per text. This Dutch version provides the full specifications of the features.

#### Word Embeddings

Word embeddings are a technique to represent word semantics on a high level (distributional semantics). It is based on the observation that words occurring in the same context [21] have similar meanings and this is captured at an abstract level by word embeddings. We applied Word2Vec (Google) [22] to implement word embeddings and mapped the vocabulary to a semantic space with 300 dimensions.

#### Document Attributes

Besides the content words in the documents, other document characteristics can also be automatically measured, namely sentence complexity, relational coherence in the text, and writing style indicators such as usage of action verbs and pronouns. Such textual characteristics could indicate emotions and writing styles may contain useful information that may indicate severity regardless of the actual content words in the text. We explored these feature types and used a list of 250 different document characteristics such as probability features including word, n-gram, and lemma frequencies; complexity features including sentence, word, and noun phrase lengths; and a wide range of other features. We computed these features with T-scan [23], a tool that was designed to predict the readability of a document.

#### Average Sentiment

We also used automatic sentiment analysis for feature representation, as we expected that severe complaints could contain more negative emotions.

Sentiment analysis is generally conducted in two ways; the first is to use a subjectivity lexicon that has annotated entries for various words, whereas the second involves classifying the documents for positive or negative sentiments using machine learning techniques. We applied the first technique and used a subjectivity lexicon for Dutch adjectives [24] to estimate the overall sentiment value per complaint based on the text description in the request field.

### Machine Learning Techniques

Given the exploratory nature of our work, we could not determine a priori which machine learning algorithm would perform the best on the data set. As is well known in the field of machine learning, and eloquently worded in the “No Free Lunch” theorem [25], there is not one machine learning algorithm that always provides the best solution. Therefore, we conducted experimental tests on the following algorithms to investigate which one was suitable for this particular task: multinomial naive Bayes, support vector machine (SVM), k-nearest neighbor (k-nn), and extreme gradient decision tree. We used the majority class baseline as the reference.

### Parameters, Evaluation Metrics, and Experimental Setup

To estimate the performance of the classifier, we trained a model on one part of the data set and evaluated the performance on a held-out sample. We used the complaints filed in the first 6 months of 2017 as the testing material and used all other complaints as the training material.

We conducted experiments to optimize the parameter settings for each of the machine learning algorithms. We performed a 10-fold cross-validation/grid search on the training set to find the optimal combination of features, algorithms, and algorithmic parameter settings. The best classifier was then applied to the test set.

Note that the complaint data set had a skewed class balance, as only 6% of the complaints were labeled “severe” when using the strict “Decision” (The inspectorate took action on the basis of the complaint) label. Machine learning techniques are known to be sensitive to such class imbalances [26].

As the evaluation metrics to estimate the performance of the classifier, we computed recall, precision, and their harmonized mean, the F1 score [27], for the severity class label. This implies that we only focused on how well we performed on the minority class label “severe.” We computed how many of the complaints that were actually labeled severe were also predicted by the system as “severe” (recall) and how many of the severity predictions were also actually labeled by humans as “severe” (precision).

As a second measure, we also computed the area under the curve-receiver operating characteristics (AUC-ROC) scores that show the balance between the true positive (TP) rate versus the false positive (FP) rate. This score also considers the predictions on the “not severe” class label.

#### Classifier Optimization

Each classifier was optimized by 10-fold cross-validation experiments on the training set. We experimented with each of the 5 different feature groups (bag of words, keywords, word embeddings, document attributes, and sentiment) individually, and each of the textual representations combined with the sentiment analysis features to investigate whether the sentiment features were predicted for severity. Lastly, we conducted an experiment combining all the feature representations. We ran grid search optimization experiments to find a suitable algorithmic parameter setting for each machine learning algorithm. We optimized the F1 score of the positive class because we were interested in a classifier that could predict the severity label.

These tuning experiments on the training set determined which classifier and which features worked best for predicting the severity of the complaints. The optimization experiments showed that the k-nn algorithm did not perform very well in this task with F1 scores of 24.5 (Triage) and 10.1 (Decision) and that the naive Bayes obtained the best results with F1 scores of 46.9 (Triage) and 32.1 (Decision).

The sentiment features did not contribute to better performance and were not helpful for severity prediction. The textual representation with the best result was the bag-of-words representation; it scored better than any of the other individual features and was also better than the combined feature representation. The classifier that performed the best on the training set (naive Bayes with the bag-of-words representation) was applied to the held-out test set. More details on the optimization experiments can be found in the supplement.

### Identifying Health Care Providers With Patient Safety Problems

We explored automatic methods to predict the severity of a complaint not only so that the Health and Youth Care Inspectorate can prioritize the most urgent complaints but also to open up possibilities for using automatic techniques that can look for patterns in the current complaints database to spot cases with elevated risk levels. As all complaints are handled using a one-by-one strategy, methods that explore the entire database can provide new insights into the data.

We also showcased how these severity predictions could be combined with keyword patterns to quickly provide insights for the inspectorate on how to extract safety problem indicators per health care provider based on the database with complaints from several years. We performed an exploratory analysis for every health care provider for which at least 10 complaints were registered in the database and used the severity predictions to rank these organizations based on their level of urgency for further inspection. The content of these grouped complaints was represented as n-grams with the most typical words and phrases so that the inspectors could identify the topics at a glance. These most descriptive n-grams per health care provider were identified using a statistical metric, log likelihood, which compares the scores of the specific terms related to health care providers with those in the entire complaints database.

## Results

### Severity Prediction

Table 1 shows the F1 values and harmonic means of precision and recall computed for the “severe” class on the held-out test set. This is a strict measurement, as we have a skewed class distribution, and the obtained F1 scores are in line with the expectations for such skewed classes. The ROC-AUC scores of 0.7 (Triage) and 0.8 (Decision) clearly indicate that the classifier performs well above chance level (random predictions lead to an AUC score of 0.5). Accuracy includes the correct predictions of the “not severe” cases, and therefore, the classifier attains high accuracy in both the labeling tasks. We can observe a slightly better score on the Triage label than the Decision label. The class distribution between severe and not severe is skewed, as only 6% of the complaints in the test set were labeled severe based on the Decision labels and 13% based on the Triage labels.

**Table 1:** Best results obtained by the naive Bayes on the test set.

Label	Accuracy	F1	Precision	Recall	AUC-ROC^a^ score
Triage	86.9	51.1	48.8	53.7	0.72
Decision	93.0	45.2	43.6	46.8	0.81

^a^AUC-ROC: area under the curve-receiver operating characteristics.

We present the confusion matrices in Table 2. Recall reflects the TP and false negative (FN), whereas precision focuses on the TP and FP. Most of the complaints are correctly labeled “not severe,” namely true negative (TN), which leads to the highly accurate scores shown in Table 1 and is also reflected in the AUC-ROC scores that consider the TN, as it reflects the TP rate against the FP rate. The skewed class distribution leads to only reasonable F1, recall, and precision scores. This is evident when observing the TP, FP, and FN values in this table. Our model overpredicts the “severe” class by mislabeling the ‘not severe’ complaints (FP) and overpredicts the “not severe” label for severe complaints (FN), leading to only a reasonable overall performance.

**Table 2:** Confusion matrices from which the scores in were computed.

Cell value		Triage		Decision	
TN^a^	FP^b^	2658	238	2990	124
FN^c^	TP^d^	196	227	109	96

^a^TN: true negative.

^b^FP: false positive.

^c^FN: false negative.

^d^TP: true positive.

### Identifying Healthcare Providers With Patient Safety Problems

Table 3 shows the top selection of (anonymous) organizations for which multiple complaints were registered in the database. We sorted these health care providers based on their average severity score, the last column in Table 3. This was computed by dividing the number of predicted severity labels (shown in the column “Severity prediction”) by the number of complaints (the second column). Note that the third and fourth columns, “Triage” and “Decision,” represent the number of complaints that were actually triaged or inspected by the inspectors. The first row of Table 3 shows that health care provider 1 received 11 complaints of which 3 were triaged, and in 2 of these 3 cases, the inspectorate took action to place the health care provider under inspection.

**Table 3:** Number of complaints and severity per organization.

Health care provider	Number of complaints	Triage	Decision	Severity prediction	Average triage	Average decision	Average severity
1	11	3	2	*9*	27.3	18.2	*81.8*
2	10	3	2	*7*	30.0	20.0	*70.0*
3	11	0	0	*7*	0.0	0.0	*63.6*
4	19	4	2	*12*	21.1	10.5	*63.2*
5	72	12	7	*45*	16.7	9.7	*62.5*
6	13	3	1	*8*	23.1	7.7	*61.5*
7	10	4	4	*6*	40.0	40.0	*60.0*
8	10	3	2	*6*	30.0	20.0	*60.0*

The anonymized version of the 10 most important (translated) terms that were identified for health care provider 1 indicate the contents of the complaints concerning perceived patient mistreatment. The top 10 word n-grams for health care provider 1 are the following: sister, scolding, safety sister, harrowing, care sister, quality life, diet, note, neglect, and employee.

## Discussion

Our study has explored whether supervised machine learning techniques can automatically determine the severity of incoming complaints. Our results showed that severity was best predicted with a straightforward text classification approach using the bag-of-words feature representation. We combined the severity predictions with word n-grams to create an overview of the most urgent complaints per individual health care provider. An overview based on the severity of the complaints could help the inspectorate in prioritizing health care providers with potential patient safety problems.

The sentiment features were not helpful in predicting the severity of the complaints. A possible explanation for this is that we are still far from achieving accurate automatic sentiment predictions [28] and that the sentiment scores were not sufficiently reliable. On the other hand, our hypothesis that severe complaints contain more negative emotions could be at fault as well. This aspect needs further investigation.

### Comparison With Other Studies

Greaves and colleagues [3] showed that text mining techniques can be used to label online patient reviews of health care providers as positive or negative, and to extract information about the opinions on aspects of care quality. Several studies [4,29,30] use text mining techniques like text classification, topic modeling, and sentiment detection on online health care provider reviews to explore which topics are indicative of perceived health care quality by patients. We focused on labeling the severity of the complaints, whereas these studies focused on patient opinions and sentiments using fine-grained doctor visit–related topics in the online reviews. An interesting path for future work would be to extend the current severity label to a more fine-grained severity label that combines severity with the type of complaint to create an automatic method similar to that established by Brereton et al [31] who performed a manual qualitative analysis of negative reviews to identify the most frequent actionable criticisms in patient reviews of hospices.

Lui et al [32] combined a data-driven topic modeling approach with expert knowledge to create a topic taxonomy of medical practitioner reviews. Their analysis of a large data set of reviews also showed that patients with different diseases focus on other topics. In our current study, we did not diversify our results for different sectors, but this would certainly be one of the aspects to investigate in future work.

Desmet [33] applied machine learning techniques to determine severity based on written text by labeling severity in social media posts related to suicidal thoughts. This study represented severity using three labels (low, intermediate, and high); SVM and k-nn algorithms were used for classification and a range of different features such as bag of words, emotion lexicons, and clustered topical words. The severity of suicidal thoughts in social media posts was predicted with an F1 score of 43% to 67%.

Our study used machine learning techniques to estimate the severity of the complaints filed with the inspectorate. The Dutch Health and Youth Care Inspectorate has already incorporated patient opinions in its supervision of health care providers. Since 2016, the ratings of Zorgkaart Nederland, the national patient rating site, are being shown in the information dashboard for inspectors, which was seen as useful extra information by inspectors [8]. In the United Kingdom, The Care Quality Commission has experimented with identifying risks and prioritizing visits by combining patient feedback from the National Health Service (NHS) Choices, Patient Opinion, Facebook, and Twitter to obtain a near real-time collective judgment score for acute hospitals and trusts on any given date [34]. This so-called Patient Voice Tracking System was successful in identifying a high-risk group of organizations for inspection.

### Implications for Practice

Our analysis can help inspectorates evaluate their own severity categorization. Moreover, the Dutch Health and Youth Care Inspectorate can use our algorithms to prioritize the incoming complaints directly based on an automatically predicted severity label. Furthermore, they can create an overview of all the complaints about a health care provider using our algorithm with an automatically predicted severity label. These labels can be used to identify soft signals [35] and blind spots [17] that indicate potential safety issues with certain health care providers. These are cases that on their own do not warrant direct action but may together indicate systematic problems. Our study produced some encouraging results, and currently, the inspectorate is investigating opportunities for using such algorithms as an assistant tool for the human experts in evaluating complaints and prioritizing visits.

Using text mining techniques such as n-grams in the set of provider-specific complaints can support prioritizing follow-up supervision activities by sorting complaints based on severity per organization or per sector. Health care inspectorates in most countries deal with several providers, and they cannot visit all the providers. Prioritizing visits based on severity prediction could improve the effectiveness of their supervision.

Predicting the severity of complaints based on algorithms can also be useful for health care providers themselves. The board of directors of the providers should be interested in knowing whether the trends of patient complaints indicate patient safety problems requiring improvement and thus prevent the inspectorate from taking action.

### Strengths and Weaknesses of This Study

One strength of this study was the collaboration with the inspectorate, as this provided a different perspective to study complaints for detecting potential safety risks, whereas most of the previous works discussed above have focused on text mining reviews to extract information on patient satisfaction. The inspectorate also provided the database containing 22,000 complaints spanning 3 years. Another strength was the use of software-supported natural language processing for Dutch. This software is part of the open-source software La Machine [36]. This software could be installed on the secure server of the inspectorate, which enabled thorough analysis. A major weakness of our study was the difficult preprocessing phase. It took months to create a secure machine learning environment that was able to host our software. For some complaints, it was difficult to automatically remove all forms of personal information.

### Implications for Further Research

In our study, we used a general LIWC list of words, which can be replaced by a specific word list created by the inspectorate. This may improve the results.

We also tried to optimize the F1 score, and the harmonic means of recall (how many relevant complaints are selected?) and precision (how many selected complaints are relevant?). For practical use, it may be more logical to optimize a severity classifier concerning completeness to select the most serious cases at the expense of precision.

For using our method in practice, a thoughtful implementation study is necessary with inspectors in the lead to optimize their support. Further, sound and up-to-date technical infrastructure for data science is indispensable for this approach.

### Conclusion

A fully automatic analysis of a complaints database with text mining techniques may support health care inspectorates in identifying potential patient safety problems at health care providers. In particular, a straightforward text classification approach using the bag-of-words feature representation can be effective for severity prediction. Using text mining techniques such as n-grams in the set of provider-specific complaints can support prioritizing follow-up supervision activities by sorting complaints based on the severity per organization or per sector.

## Abbreviations

AUC-ROC: area under the curve-receiver operating characteristics
FN: false negative
FP: false positive
HCAT: Healthcare Complaints Analysis Tool
k-nn: k-nearest neighbor
LIWC: Linguistic Inquiry and Word Count
NHS: National Health Service
SVM: support vector machine
TN: true negative
TP: true positive
